# The Potential Impacts of Urban and Transit Planning Scenarios for 2031 on Car Use and Active Transportation in a Metropolitan Area

**DOI:** 10.3390/ijerph17145061

**Published:** 2020-07-14

**Authors:** Patrick Morency, Céline Plante, Anne-Sophie Dubé, Sophie Goudreau, Catherine Morency, Pierre-Léo Bourbonnais, Naveen Eluru, Louis-François Tétreault, Marianne Hatzopoulou, Naveen Chandra Iraganaboina, Tanmoy Bhowmik, Audrey Smargiassi

**Affiliations:** 1 Direction de Santé Publique du Centre Intégré Universitaire de Santé et de Services Sociaux du Centre-Sud-de-l’Île-de-Montréal, Montreal, QC H2L 1M3, Canada; patrick.morency@umontreal.ca (P.M.); celine.plante.ccsmtl@ssss.gouv.qc.ca (C.P.); anne-sophie.dube.ccsmtl@ssss.gouv.qc.ca (A.-S.D.); sophie.goudreau.ccsmtl@ssss.gouv.qc.ca (S.G.); louis-francois.tetreault.ccsmtl@ssss.gouv.qc.ca (L.F.T.); 2 Département de Médecine Sociale et Préventive, École de Santé Publique (ESPUM), Université de Montréal, Montreal, QC H3N 1X9, Canada; 3 Department of Civil, Geological and Mining Engineering, Polytechnique Montreal, Montreal, QC H3T 1J4, Canada; cmorency@polymtl.ca (C.M.); leo.bourbonnais@polymtl.ca (P.-L.B.); 4 Department of Civil, Environmental and Construction Engineering, University of Central Florida, Orlando, FL 32816, USA; Naveen.Eluru@ucf.edu (N.E.); naveen.chandra@Knights.ucf.edu (N.C.I.); tanmoy78@Knights.ucf.edu (T.B.); 5 Department of Civil & Mineral Engineering, University of Toronto, Toronto, ON M5S 1A4, Canada; marianne.hatzopoulou@utoronto.ca; 6 Institut National de Santé Publique du Québec, Montreal, QC H2P 1E2, Canada; 7 School of Public Health, Centre of Public Health Research, University of Montreal and CIUSSS du Centre-Sud-de-l’Île-de-Montréal, Montréal, QC H3T 1A8, Canada

**Keywords:** urban growth scenario, sprawl, transit-oriented development, modal share, public transit, car use, active transportation, walking

## Abstract

Land use and transportation scenarios can help evaluate the potential impacts of urban compact or transit-oriented development (TOD). Future scenarios have been based on hypothetical developments or strategic planning but both have rarely been compared. We developed scenarios for an entire metropolitan area (Montreal, Canada) based on current strategic planning documents and contrasted their potential impacts on car use and active transportation with those of hypothetical scenarios. We collected and analyzed available urban planning documents and obtained key stakeholders’ appreciation of transportation projects on their likelihood of implementation. We allocated 2006–2031 population growth according to recent trends (Business As Usual, BAU) or alternative scenarios (current planning; all in TOD areas; all in central zone). A large-scale and representative Origin-Destination Household Travel Survey was used to measure travel behavior. To estimate distances travelled by mode, in 2031, we used a mode choice model and a simpler method based on the 2008 modal share across population strata. Compared to the BAU, the scenario that allocated all the new population in already dense areas and that also included numerous public transit projects (unlikely to be implemented in 2031), was associated with greatest impacts. Nonetheless such major changes had relatively minor impacts, inducing at most a 15% reduction in distances travel by car and a 28% increase in distances walked, compared to a BAU. Strategies that directly target the reduction of car use, not considered in the scenarios assessed, may be necessary to induce substantial changes in a metropolitan area.

## 1. Introduction

Many health benefits and adverse impacts are associated with current urban transportation systems. Transportation related public health problems such as crashes, noise and air pollution have been quantified as functions of traffic volume or associated to the proximity to major roads [1]. Kilometers travelled by car induce public health problems to people living in locations along car trips (e.g., arteries, through traffic) [2]. Car use has been associated with physical inactivity and related health problems, while public transit (PT) use requires an amount of walking which helps achieve the recommended level of daily physical activity [3,4,5,6]. However, PT may not be optimal due to travel discomfort and fixed schedules and can also contribute to exposure to pollutants.

In most North American cities, tram systems were abandoned in the late 40s (1959 in Montréal, Canada) to give way to buses and trolleybuses [7]. The concurrent development of a network of major road infrastructures (e.g., highways, arteries) enabled commuters to live far from urban centers, in areas with poor access to PT and few services. For example, from 1961 to 2006, the demographic weight of the Montreal Island within the Montreal Metropolitan Community (MMC) declined from 78% (1961) to 52% (2006) [8] and, nowadays, the peripheral municipalities of Montreal—outside the MMC—are among the fastest growing in Canada [9]. Nowadays, in Montreal, trips made by car represent ~70% of all morning peak trips and, from 2008 to 2013, the private car fleet increase was two times greater than the population [10]. Although urban sprawl is multidimensional [11,12], at the metropolitan level, low population and employment density has been strongly associated with increased car use and vehicle-kilometer travelled (VKT) instead of active transportation [13].

Compact development and transit-oriented development (TOD) are comprehensive responses to the challenges raised by automobile dependency and urban sprawl [11,14,15]. Regarding the TOD concept, there are no consensus and various subtypes have been defined—pedestrian-oriented vs. transfer oriented; regional vs. neighborhood TOD; transit-adjacent vs. transit-oriented development; and so forth. [7,16,17]. Nevertheless, the principles are well known and generally include a mix land-use (residential and commercial), a certain level of density and a walking distance to a transit station and core commercial area. The spatial scale is also relevant, since potential interventions can target an entire metropolitan area (e.g., densification, transportation networks), specific transport corridors, neighborhoods or specific urban lots and streets. To address public health problems at the population level, a macro-level perspective (e.g., metropolitan) is appropriate.

Over the last thirty years, many North American cities tried to integrate the concept of TOD in their urban planning. Montreal (Canada) is no exception [18]. Meanwhile (also in the 90s), land use-transportation scenario planning practice emerged [19,20,21] and provided opportunities to evaluate alternative development patterns and analyze their impacts on, for example, urban sprawl [22,23,24,25], regional VKT by car [19], the environment (e.g., air pollution and green house gases (GHG)) [26,27]; and health [14,28,29,30]. In these studies, future scenarios have mostly been based on hypothetical/theoretical development or have assessed planning visions. Very few studies have assessed the impacts of scenarios based on official planning documents based on stakeholders views even though Avin and Goodspeed (2020) [31] suggested that to inform urban planning, scenarios should not only rely on expert judgement but on stakeholder values. Furthermore the impacts of such scenarios have rarely been compared to more exploratory scenarios.

Our main objective was to contrast the impacts on car use and active transportation in a metropolitan area (Montreal, QC, Canada), of a land use and transportation scenario based on current strategic planning documents and key stakeholders views, with impacts of hypothetical and extreme scenarios.

## 2. Methods

The Methods section includes the steps taken to develop the scenarios, then a description of the scenarios and of the approaches used to estimate the modal share and distances travelled in 2031 for each scenario.

### 2.1. Development of 2031 Scenarios

**Figure 1:**
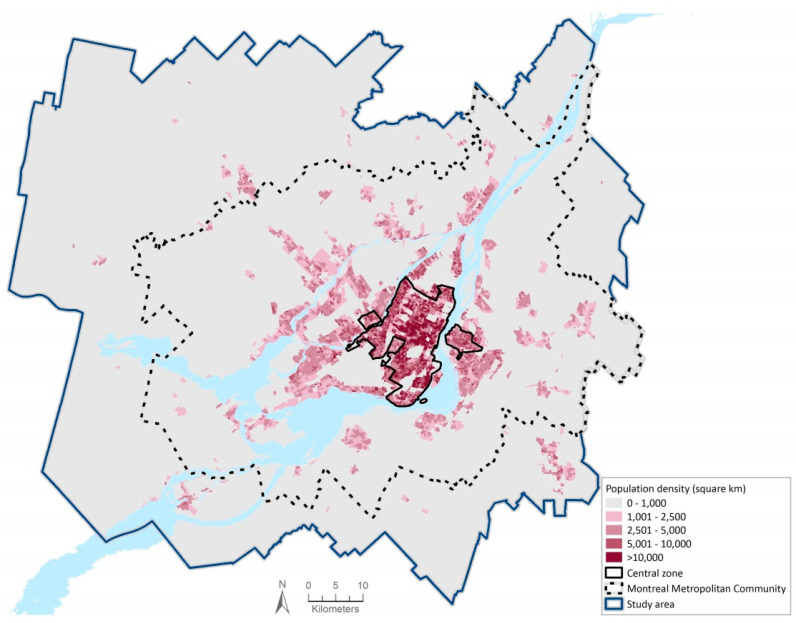
Population density in the Greater Montreal Area (2006).

To cover the peripheral municipalities of Montreal, we used the Greater Montreal Area (8327 km^2^; see Figure 1) defined for transportation planning purposes [32] instead of an administrative delimitation such as the Montreal Metropolitan Community (MMC; 3838 km^2^) or the Montreal’s Census Metropolitan Area (MMA; 4258 km^2^). In 2006, the Greater Montreal Area had a population of 3,939,834, divided in 108 municipal sectors [32] or, for population estimation at the local level [33], in 216 “Planning Zones.” These “Planning Zones,” first defined in the ‘90s, correspond to entire municipalities or smaller areas with specific development challenges or opportunities (e.g., historic Old Port of Montreal, former industrial sectors).

We developed a strategic planning scenario based on following information and discussions with professionals and stakeholders. We collected and analyzed available urban planning documents at the municipal and regional levels. In 2012, the Montreal Metropolitan Community (MMC) adopted a strategic land use and transportation plan for the 2011–2031 period—the Metropolitan Land Use and Development Plan (PMAD: “Plan métropolitain d’aménagement et de développement”) [34]. The PMAD was found to be the most recent, comprehensive planning process and the only one which covered most (94%) of the Greater Montreal Area population. Furthermore, in 2012 the PMAD was seen as a breakthrough, because it emphasized the need for density and included 82 municipalities with conflicting interests. The PMAD identified existing or potential TOD areas, delimited by a 1 km radius surrounding a high capacity PT access [34]. It targeted 98 km^2^ of land available for residential development and defined minimal density thresholds which varied according to distance from downtown and, within TODs, according to the type of PT (e.g., subway, train or bus); it also included new PT projects (see Section 2.2).

Throughout our review of planning documents, we identified potential transportation infrastructure projects for the Greater Montreal Area (e.g., roadway extension, new PT, park and ride lots, etc.). In January 2017, we surveyed 23 professionals and stakeholders from municipalities, urban or transportation planning agencies and NGOs to obtain their appreciation of Greater Montreal Area planning and projects. PT projects were considered “likely” to be implemented if the majority of participants thought that the likelihood of implementation was 50% or more by 2031; they included all the PMAD PT projects. As suggested by the participants, we defined a “central zone” not limited to the Central Business District of the island of Montreal but instead based on current residential density (Figure 1). The “central zone” includes 32 contiguous “Planning Zones” with a gross residential density greater than 20 dwelling units per hectare and covers an area of 235 km^2^.

### 2.2. Description of Scenarios

**Figure 2:**
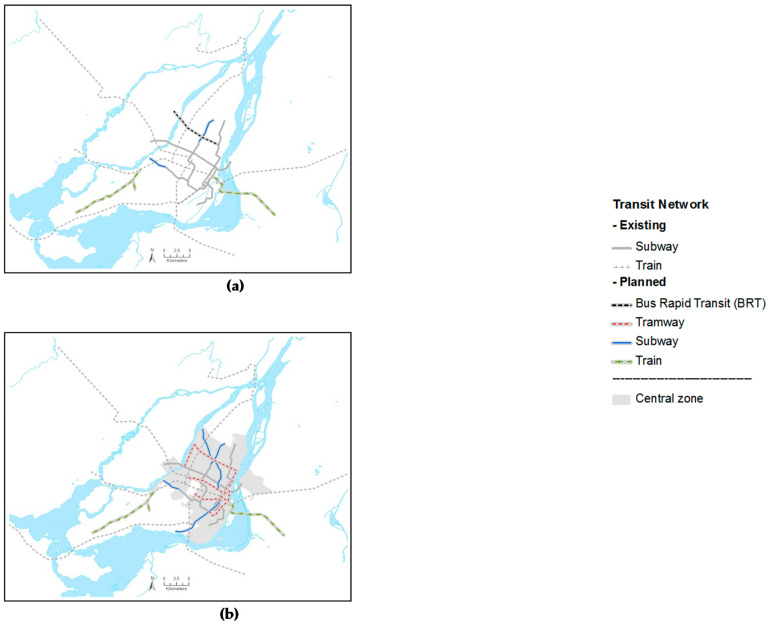
Future public transit infrastructures associated with 2031 alternative scenarios; (**a**) Planned and transit-oriented development (TOD)100% scenarios; (**b**) Central scenario.

Table 1 briefly describes the main objective and the allocation of population growth for each scenario. The 2031 Business As Usual (BAU) scenario is based on past socio-demographic trends and serves as the reference to which other scenarios are compared. No PT infrastructure is added to the BAU (Figure 2a), which includes 122 train or subway stations. In this scenario, the households added between 2006 and 2031 are distributed among all 216 “Planning Zones” according to previously observed migration patterns (1996–2006) and without any household capacity constraints.

Aside from the BAU scenario and the scenario based on current planning (referred to as the planned or PMAD scenario), we included two additional scenarios which increase the population living in TODs or in the “central zone” (Table 1). All include planned transportation infrastructures (i.e., those of the PMAD described below) while population distribution of the population growth varies.

The “Planned” scenario is based on the PMAD strategic planning document to represent the current urban planning at the Greater Montreal Area level. Its objectives aim (i) to maintain on the island of Montreal a significant proportion (38.5%) of the population growth and (ii) to locate in TOD areas 36.8% of all new households in the Greater Montreal Area. Two subway extensions, a bus rapid transit (BRT) line and a new light rail transit were considered likely to be built in 2031 and are included (Figure 2a), adding 21 stations and potential new TODs to the Greater Montreal Area. It is a realistic scenario.

The two other scenarios modelled different ways to increase the population in regions with high capacity PT availability. The “TOD 100%” scenario allocates all the population growth (not only 36.8%) to TOD areas identified in the “Planned (PMAD)” scenario to explore their maximal potential contribution (without household capacity constraints). Population growth was also allocated in the existing TODs. Otherwise, the PT projects (Figure 2a) were similar to the “Planned (PMAD)” scenario. The “Planned” scenario but even more the “TOD100%” scenario can be viewed as favoring a “stellar shape” development.

The « Central » scenario aims to concentrate the population growth within neighborhoods with pre-existing high population density, previously defined as the “central zone” (Figure 1). This scenario explores the potential impacts of allocating population growth in the densest regions of the Greater Montreal Area, with substantial addition of PT. Major PT projects unlikely to be implemented in 2031 but located in the central zone, for example, tramways (59 stations), a new subway line under current public debate (29 stations) were included, in addition to those of the PMAD (Figure 2b). This results in 272 PT stations for this scenario compared to 122 in the BAU and 143 in the “Planned” and “TOD100%” scenarios. Thus, compared to all other scenarios, a greater proportion of the population was located in the central zone (Table 1) where there are more PT services.

### 2.3. Estimation of Population, Modal Share and Distances Travelled for 2031 Scenarios

This section describes the data sources and the methodology to estimate population numbers, modes and kilometers travelled that would result from the scenarios.

The Institut de la statistique du Québec (ISQ) projected that 498,721 new households and 871,052 residents will be added to the Greater Montreal Area from 2006 to 2031 (ISQ: Scenario A, éd. 2011). The predicted population increase in the Greater Montreal Area over this 25-year period (+ 34,842 residents per year) was slightly lower than the actual annual increase from 2006 to 2016 (+ 38,768 per year) (source: 2006 & 2016 Canadian censuses). We used a tool/software (ES-3) developed and used by the Quebec ministry of transportation for more than 20 years to allocate future households across all the “Planning Zones” of the province of Quebec, including 216 “Planning Zones” in the Greater Montreal Area [33]. ES-3 allocates households according to recent migration patterns observed in past censuses, both within the province of Quebec and within the Greater Montreal Area. It takes into account, the “Planning Zones” characteristics (e.g., age and sex of migrants, maternal language, access to PT, etc.), birth and mortality rate and their future households’ capacity. Household’s capacity in 2031 was modified according to each scenario’s targets and specifications.

We also used the 2008 origin-destination survey (O-D survey), a large scale representative travel survey of 66,100 Greater Montreal Area households which collected data on all household individuals and their previous weekday trips (*n* = 322,800 trips). The O-D surveys provided information at the household level (location, car ownership, etc.), household members (age, sex, driver’s license, number of trips, etc.) and trips of household members (origin, destination, departure time, motive, mode, etc.). This survey includes individual and trip weights—related to sampling fractions—calculated to be representative of the 2006 Greater Montreal Area population. Appropriate data from more recent O-D surveys were not available when this project was completed.

The expected population and trips in 2031 were estimated using the O-D individual weight (OD2008WEIGHT) multiplied by three expansion factors related to population increase (POP2031), motorization (CAR2031) and workforce trends (ACTIVITY2031) from 2006 to 2031. First, we assigned future household capacity within the Greater Montreal Area, according to each scenario’s targets and specifications. This input was used by ES-3 to calculate, for each scenario, a population expansion factor at the “Planning Zone” level, for each age-sex stratum (POP2031 = population in 2031/population in 2006). Second, expected 2006–2031 trends in motorization (increase among women and students; CAR2031) and in the activity status (aging workforce, more women; ACTIVITY2031) were taken into account with specific age-sex expansion factors that were already calculated at the Greater Montreal Area subregion-level and provided by the Ministry of transportation [35]. Third, we corrected small discrepancies between scenarios (i.e., distribution per age, sex and activity status) attributable to geographical variation in household characteristics through a post-stratification of the 2031 individual weights by age, sex and activity status, taking as reference the BAU scenario. Fourth, for scenarios which increased the population in specific areas with few O-D participants (new peripheral TODs) where the 2031 weights could not be applied, we implemented a procedure (clones) detailed in Appendix A.

We predicted transportation modes in 2031 using two methods. We used mode choice models based on travel time and cost, individual and household attributes [36,37]. We also used a simpler method, to predict transportation modes in 2031 that was more sensitive to mode shift; it was based on the 2008 mode shares in the Greater Montreal Area population: 216 strata were defined based on individual characteristics (age, sex), household location (central or not, within a TOD area or not) and trip mode (car, PT, walk and bicycle). For each stratum k, the number of trips in 2031 was calculated according to Equation (1). Then, for each scenario, the initial 2008 modal shares within each population stratum were applied to the 2031 population strata to calculate the number of trips per mode in 2031. Other trip characteristics (e.g., origin-destination) were kept similar to 2008.

(1) $$NTrips2031_{k}=\overset{n}{\underset{i=1}{\sum }}\left(NTrips2008_{i}\;\times \;Weight2031_{i}\right)\;$$

where

*i* = individual of the stratum *k*
*k* = one of the 216 strata
*n* = number of individuals in the stratum *k*
NTrips2008i = number of trips in 2008 of individual *i*, all modes included.
NTrips2031k = number of trips in 2031 for the population in stratum *k*
Weight2031i = O-D survey individual weight adjusted for 2031, as described above (OD2008WEIGHT × POP2031 × CAR2031 × ACTIVITY2031 (described in text above).

## 3. Results

In 2006, 26% of the Greater Montreal Area population was living within TOD areas, that is, less than one km from a train or subway station and 37% was living in the “central zone” (Table 2). The average gross population density within TOD areas was four times greater in the central zone than in the periphery.

### 3.1. Impacts of Scenarios: Population Distribution

**Figure 3:**
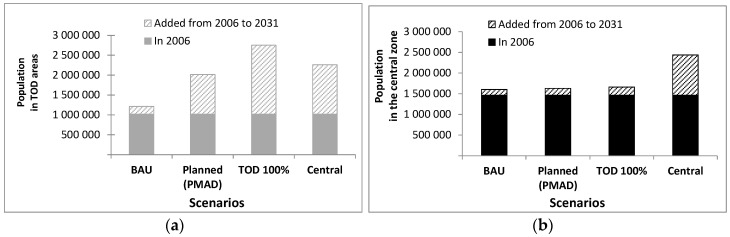
Population distribution, according to 2031 scenarios; (**a**) Population living in TOD areas; (**b**) Population living in the central zone.

The population growth allocation within the Greater Montreal Area from 2006 to 2031 is shown in Table 2 and Figure 3. In 2031, the population living in a TOD area (i.e., within one km from a train or subway station) was greatest in the TOD100% scenario (57% vs. 47% in Central, 42% in Planned, 25% in BAU scenarios; Figure 3a). The population living in the “central zone” was, obviously, greatest in the Central scenario (51%) than in other scenarios (from 33% to 35%) (Figure 3b). However the Central scenario included only ~10% less or individuals in TOD areas than the TOD100% scenarios.

Overall, in the Greater Montreal Area, from 2006 to 2031, there was a 22% increase in population density, from 474 to 578 people/km^2^ (Table 2). Compared to the BAU, the greatest increase in density was achieved with the TOD100% scenario, within the TODs located in the periphery (+280%; 8, 250 persons/km^2^ in 2031) but the greatest density in 2031 was achieved with the Central scenario, within TODs in the “central zone” (+48%; 11,737 persons/km^2^ in 2031).

### 3.2. Impacts of Scenarios: Transportation Modes and Distances Travelled

**Figure 4:**
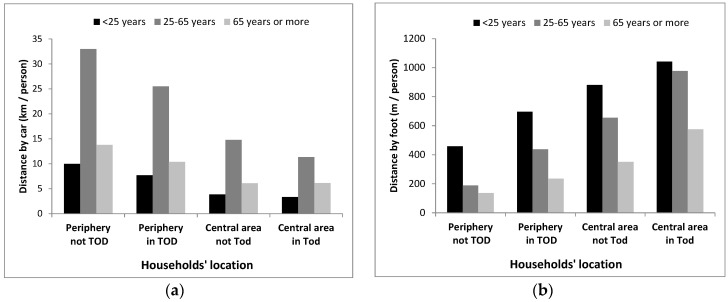
Average daily distance travelled (according to the method based on 2008 mode shares), by age group and households’ location (business as usual (BAU), 2031); (**a**) By car; (**b**) By foot (walk alone or for public transit (PT)).

Based on the method to estimate mode shift with 2008 mode shares, in the BAU scenario, car use was much lower in the “central zone” than in the periphery, for all age groups (Figure 4a). The average daily walking was greater for people living within the central zone than in the periphery and for people living in TOD than outside TOD areas, for all age groups (Figure 4b). Younger populations walked more for travel while the age group 25–65 years contributed the most to distances travelled by car.

**Figure 5:**
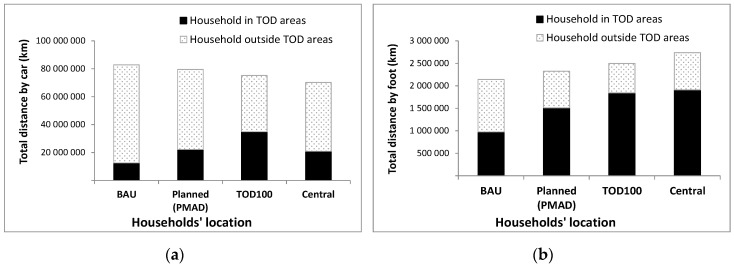
Total kilometers travelled (according to the method based on 2008 mode shares), across scenarios and by households’ location (Greater Montreal Area, 2031) (**a**) By car; (**b**) By foot (walk alone or for PT).

Overall in 2031, for the entire Greater Montreal Area population, there was a transfer from car use to active and public transportation—measured by the daily number of trips—in all three alternative scenarios, when using the method based on the 2008 mode shares (Table 3). Compared to the BAU, there were slight reductions in the average distances travelled per car trip in alternative scenarios and in distance travelled by PT trip in the Planned (PMAD) and Central scenarios. Compared to the BAU, the estimated reduction in the total kilometers travelled by car (as driver or passenger) was greater in the Central (−15%) than in the TOD100% (−9%) or in the Planned scenario (−4%) (Table 3 and Figure 5a). Compared to BAU, the estimated increase in the overall distances walked (including trips solely by foot or with PT) was greater in the Central scenario (+28%) than in the TOD100% (+16%) or the Planned (+8%) scenarios (Table 3 & Figure 5b).

Figure 5 shows the total distances travelled by car or walked in 2031 across scenarios (based on the method to estimate mode shift with 2008 mode shares), according to household location. The TOD100% scenario—which allocated all the population growth in existing and potential TOD areas—greatly increased the proportion of Greater Montreal Area’s car travel generated by household located in TOD areas (15% in BAU; 28% in Planned; 30% in Central; 47% in TOD100%). Compared to the BAU, all three alternative scenarios increased the proportion of Greater Montreal Area’s kilometers walked generated by household located in TOD areas (46% in BAU; 65% in Planned; 70% in Central; 74% in TOD100%).

Compared to results based on the method to estimate mode shift with 2008 mode shares presented above, there were very little and even counterintuitive changes in mode shift based on the mode choice model; predicted times were also similar for all scenarios (see Appendix B).

## 4. Discussion

We contrasted future impacts, on transportation modes and km travelled, of a scenario based on strategic planning documents, with those of hypothetical scenarios. Compared to the BAU, the scenario based on strategic planning representing the current Greater Montreal Area targets for 2031 only marginally influenced trips and distances travelled by car. The impacts on transportation behavior were greatest in the scenario that allocated population growth to already dense areas (Central scenario) and that also included numerous PT projects unlikely to be implemented in 2031 (e.g., tramways, a new subway line). Such major changes would nonetheless lead to relatively minor impacts in the Greater Montreal Area, inducing at most a 15% reduction in distances travel by car and a 28% increase in distances walked, compared to a BAU. Impacts were even less when using a mode choice model, instead of past mode shares for calculations.

Our scenarios support that the location of the population influences the distances travelled by car. Identical PT and overall population density for the planned and the TOD100% scenarios induced a 9% decrease in VKT when the new population was entirely constrained to TOD areas (TOD100%) and to a 4% decrease in VKT when it was not (planned scenario). Whether population density could have a greater influence than its location cannot be addressed by our study but according to Ewing [13], “the distribution of population and employment might be more important than overall density at the regional level.”

The minor impacts of our scenarios on distances travelled by car in the metropolitan area are not surprising. Nonetheless the minor impacts we observed are higher than those that would have been obtained based on elasticity of VMT with respect to regional density as reported by Bartholomew and Ewing 2008 [38] (−0.075 VMT for every 1% increase in population density). Indeed based on that measure, we would have noted a ~1.5% decrease instead of a 4% decrease as noted for our BAU. Their elasticity measure was based on studies from the United States which may partly explain this difference. In any case this decrease is small and as documented by others [11,20,34,39], urban planning strategies based on investments in PT with populations allocated in their proximity are associated with modest reductions in car travel. The combination of many strategies, including not only PT investments but also the reduction of road capacity and other incentives to reduce car use (e.g., price of car ownership and travel), may be necessary to induce changes that are more important for a metropolis [11,20].

Strategies targeting the reduction of road capacity and car use may also be necessary to induce transportation mode changes when using a mode choice model. This may explain why our mode choice model and its travel times were insensitive to the location of the new population and to PT investments (predicting accurate travel times by mode is a challenge worth noting). These results are in line with those of Wang et al. (2016) [39] who also noted minimal car mode changes with TOD policy scenarios using a mode choice model. However, predictions by mode choice models may be poor in local TOD areas, while globally predictions may be reasonable.

Distances travelled by populations from TOD areas were more influenced by our scenarios than those from the entire metropolitan area. When considering only TOD areas, distances walked increased from ~1 M km in the TOD areas of the BAU to up to ~1.9 M km in the TOD areas of the Central scenario. The greater distances walked in the TOD areas and more specifically in the urban zone of the Central scenario are not surprising, as the new population was given the same transport behavior as the initial inhabitants of the high-density areas where such behaviors have been well documented [40].

However, compared to the planned scenario (PMAD) we noted higher km travelled by cars by populations from TOD areas in the scenario that favored their densification (TOD100% with “stellar shape” development) which is worth noting. This could illustrate the “paradox of densification” where more people means more cars, whilst the aim of densification is the reduction of car use [41]. Nonetheless, this trend was not seen with the scenario densifying the already dense central urban core and that included more PT infrastructure. Thus, km travelled by cars by populations from TOD areas likely reflect the fact that those living in TOD areas in the periphery of the urban core are still using their cars (assuming behaviors remain the same as in 2008 in TOD areas in the periphery). In the Greater Montreal Area periphery, the surroundings of most PT stations look more like “transit-adjacent” (TAD) than “transit-oriented” (TOD) development [17]. Previously published scenarios, developed for a medium-sized city in France, also showed the limits of densification around PT stations where the transit supply is not sufficient to compete with car travel [23].

A strength of our study is that our scenarios were based on disaggregated data. Furthermore, one of the scenarios was based on strategic planning documents for the metropolitan area which grounds the study in reality. While the implementation of this scenario is highly probable, a limitation of our work is that we are unable to explain how the hypothetical scenarios could be achieved. We assumed for instance that the Greater Montreal Area could reach higher densities, yet it is more difficult to modify the density of an area once developed than an unbuilt area. Even with the most realistic scenario, when important stakes are at hand, unwanted developments may occur in zones where according to plans they should not occur. Furthermore, the impacts of our scenarios are limited by the fact that they did not include constraints on car use as discussed above. Additionally, the PT components included trains, subways, tramways and one BRT line but did not otherwise improve the quality, level and coverage of PT service. We also ignored the probable development of autonomously driven buses, taxis and cars and the likely increase in teleworking. And land use scenarios do not consider potential disadvantages of living in dense areas (e.g., living in proximity and infectious disease propagation). It is also worth noting that the estimated trends from 2006 to 2016 in population distribution were conservative—according to census data, population growth has occurred more outside the potential TOD areas than predicted by our BAU scenario. We also did not consider that in 2031, car share could be lower (and PT share higher) than in 2008 in TOD areas (we applied 2008 TOD mode shares for the future), especially in the suburbs.

Notwithstanding all these limits, our scenarios remain useful to orient urban development and transportation policies. Future work can address limits of our study and also clarify which demographic groups will most likely change their modes according to various scenarios.

## 5. Conclusions

We developed future scenarios for a large metropolitan area, based on urban planning and PT projects and quantified their potential impacts on car use and active transportation. The current PT projects in the Greater Montreal Area as well as our hypothetical scenarios seem insufficient to induce a modal shift, perhaps because they lacked important strategies to reduce car use, such as road pricing or limiting road capacity. Future research should include and modify components of scenarios related to car use.

## Figures and Tables

**Table 1:** Scenarios’ main objective and allocation of new population (2006–2031).

		REFERENCE (BAU)	PLANNED (PMAD)	TOD 100%	CENTRAL
**Objective**		Projection of recent trends	Implementation of current planning (PMAD) ^1,2^	Concentration of new households in TOD areas ^2^	Concentration of new household in central neighborhoods
**Allocation of New Population (2006–2031)**	Targeted development area	All Greater Montreal Area	All Greater Montreal Area, including 143 TOD areas	Limited to 143 TOD areas	Limited to central zone ^4^
	Estimation of household capacity at the local level	Unlimited	Based on land area available for residential development and on minimal household density thresholds (PMAD) ^3^		Unlimited
	Allocation across Greater Montreal Area subregions	Based on past trends (1996–2006)	According to PMAD (39% on the island of Montreal, 21% in suburbs, 40% in outskirts)		100% in central zone
	Allocation across planning zones		Proportional to household capacity		Based on recent trends (2007–2015)
	Allocation within and outside TOD areas	N/A	36.8% located in TOD areas ^5^	100% located in TOD areas	N/A

N/A: Not applicable. ^1^. Adopted in 2012, the “Plan métropolitain d’aménagement et de développement” (PMAD) is a strategic land use and transportation plan for the 2011–2031 period. ^2^. TOD areas were delimited by a 1 km radius surrounding a train, light train, subway or bus rapid transit station; 143 TOD areas (122 existing and 21 new ones) were considered in the Planned and TOD100% scenarios; “stellar shape” development. ^3^. Minimal density thresholds defined in the PMAD were used. Expected density was greater in the central zone (vs periphery), within a TOD (vs outside TOD area) and surrounding a subway station (vs train or bus rapid transit). ^4^. The central zone (235 km^2^) includes 32 planning zones with a gross residential density greater than 20 dwelling units per hectare in 2006. ^5^. To achieve the overall target of 36.8% of population growth located in TOD in the entire Metropolitan Montreal Community, the PMAD specified targets for 3 subregions: 73.2% on Montreal island, 18.4% in the suburbs, 11.6% in the outskirts.

**Table 2:** Travel behavior, population distribution and density ^1^ according to residential location in the Greater Montreal Area.

			Households in the Central Zone		Households in the Periphery		GMA
			In TOD ^4^	Not TOD	In TOD ^4^	Not TOD	
**Context**	Population (2006)		810,145	651,555	210,530	2,274,979	3,947,210
	Gross population density (2006)		7415	5173	1856	285	474
	Daily trips per capita ^2^	Car ^3^	0.90	1.11	1.45	1.54	1.33
		Public transit	0.64	0.50	0.27	0.15	0.32
		Walk	0.45	0.31	0.20	0.13	0.23
		Bicycle	0.06	0.03	0.03	0.02	0.03
	Distance per trip (km) ^2^	Car ^3^	9.38	9.23	12.47	15.34	13.53
		Public transit	6.82	8.21	15.81	16.39	10.07
		Walk (alone)	0.90	0.84	0.96	0.93	0.89
		Walk (to/from PT)	0.84	0.82	1.09	0.97	0.90
		Bicycle	3.62	4.15	3.73	3.39	3.66
**Scenarios (2031)**	Population	BAU	921,817	680,483	296,086	2,912,499	4,810,886
		Planned	1,331,995	296,732	684,303	2,497,855	4,810,886
		TOD100%	1,290,833	370,093	1,460,292	1,689,669	4,810,886
		Central	1,932,016	504,382	330,089	2,044,399	4,810,886
	Gross population density (/km^2^)	BAU	7912	5732	2169	366	578
		Planned	10,348	2786	3866	316	578
		TOD100%	10,028	3475	8250	213	578
		Central	11,737	7143	1788	259	578

^1^ Gross population density was calculated by dividing the population numbers per the TOD and non-TOD areas, which excluded water bodies; agricultural land was also excluded for a few TOD areas. ^2^ Travel behavior measured from 2008 Origin-Destination survey; grouped by people living in the central zone or the periphery. ^3^ Includes car travel as driver or passenger. ^4^ The number of TOD areas varies across scenarios (see Methods section).

**Table 3:** Daily number of trips and distances travelled by mode (according to the method based on 2008 mode shares), for the 2031 scenarios for the Greater Montreal Area.

		2031 Scenarios			
		BAU	Planned	TOD100%	Central
**Trip/capita**	All Modes	1.97	1.97	1.98	1.98
	Car (drivers)	1.04	1.01	0.99	0.93
	Car (pass.)	0.27	0.26	0.26	0.26
	Public transit	0.29	0.31	0.33	0.37
	Walk	0.22	0.23	0.24	0.28
	Bicycle	0.03	0.03	0.03	0.03
	Other	0.13	0.13	0.12	0.11
**Average Km/trip**	All Modes	10.57	10.31	9.96	9.43
	Car (drivers)	13.95	13.73	13.16	13.02
	Car (pass.)	10.77	10.64	10.13	10.19
	Public transit	10.23	10.03	10.29	8.71
	Walk (alone)	0.90	0.90	0.91	0.89
	Walk to/from PT	0.88	0.89	0.91	0.86
	Bicycle	3.65	3.62	3.65	3.63
**Total km travelled in the GMA**	All Modes	100,096,247	97,614,291	94,688,314	89,684,927
	Car (drivers)	69,507,918	66,457,283	62,606,951	58,144,535
	Car (pass.)	13,953,653	13,557,560	12,887,231	12,516,719
	Public transit	14,031,262	14,771,132	16,162,827	15,686,491
	Walk (alone)	938,496	1,017,331	1,067,909	1,191,138
	Walk to/from PT	1,210,422	1,313,245	1,428,951	1,555,687
	Bicycle	454,496	497,739	534,444	590,357

## Appendix A. Population Growth in Peripheral Transit Oriented Development (TOD) (“Clones”)

For scenarios which increased the population in specific areas with few Origin-Destination (O-D) participants (e.g., new peripheral TOD areas targeted in Planned & TOD100% scenarios), the 2006–2031 population expansion factors (ratio of 2031 weight to 2006 weight) could not simply be applied to the original O-D survey weight. Therefore, for scenarios adding new TOD areas (Planned, TOD100%), all O-D participants living in a “Planning Zone” with a TOD area (and their trips) were duplicated: A clone living within the TOD area (“Clone-In-TOD”) and a clone living outside the TOD areas (“Clone-Outside-TOD”). So, in the O-D dataset, the trips of Clone-In-TOD were assigned a part of the 2031 individual weight (the fraction in TOD area) while the trips of Clone-Outside-TOD were assigned the rest of the weight (1—weight of Clone-In-TOD). Household coordinates of the Clone-In-TOD were changed to randomly allocated coordinates within the TOD area. In this process, we changed motor-vehicle trips coordinates if their origin or destination was the residence but, otherwise, trips coordinates were left unchanged. Walking and bicycle trips O-D coordinates were left unchanged, because it could have given unrealistic distances for active transportation. Other characteristics of individuals (e.g., age, number of daily trips) were kept identical. This process made it possible to modify the population size in TOD areas (weight of Clone-In-TOD) to meet the targets of the Planned and TOD100% scenarios. For the Business As Usual (BAU) and Central scenarios, the 2006–2031 population expansion factors were applied to the original 2006 O-D weight.

## Appendix B. Mode Choice Models

Trip-level mode choice models were developed, based on 2008 O-D survey and 2013 roadway and public transit infrastructures and applied to the 2031 scenarios [35,36]. The models took into account travel time and cost, individual and household attributes, as well as the 2031 individual’s weight specific to each scenario. For all scenarios, car ownership was based on predicted trends for 2006–2031 (see CAR2031 expansion factor in main text). In the BAU scenario, the road network and Public Transit (PT) infrastructure were considered to be similar to 2013 but, in alternative scenarios, the estimated travel time in public transit took into account all the PT infrastructures added for each specific scenario.

At the trip-level, according to the mode choice models, the alternative scenarios did not induce a modal shift from cars toward public transit compared to the BAU (see Table A1). For example, car use represented a similar mode share across scenarios for the entire Greater Montreal Area (from 66.1% to 67.4%), for households located in the central zone (from 52.3% to 53.1%) and for households located in the periphery (from 73.9% to 75.2%).

Table A2 considered the 2031 weighted trips, which are specific to each scenario and represent the entire 2031 Greater Montreal Area population. Compared to the mode choice models, our simpler method (described in Section 2.3) might have slightly underestimated car use in the alternative scenarios and, accordingly, overestimated transit use and active transportation.

**Table A1:** Modal share for each 2031 scenario, according to mode choice analyses, for the entire Greater Montreal Area, the “central zone” and the periphery (trips unweighted) *.

		Car	Public Transit	Walk	Bicycle	Bimodal **	Other
All Greater Montreal Area	BAU	66.6%	14.3%	10.8%	1.2%	1.7%	5.5%
	PMAD	66.1%	14.6%	10.9%	1.3%	1.7%	5.4%
	TOD100%	66.5%	14.2%	10.7%	1.2%	1.7%	5.6%
	Central	67.4%	13.6%	10.6%	1.2%	1.6%	5.5%
Central zone	BAU	52.3%	25.2%	16.9%	1.5%	1.5%	2.7%
	PMAD	52.7%	24.6%	16.8%	1.5%	1.5%	2.9%
	TOD100%	53.1%	24.4%	16.8%	1.4%	1.4%	2.9%
	Central	52.3%	25.1%	16.8%	1.5%	1.5%	2.8%
Periphery	BAU	74.6%	8.1%	7.3%	1.1%	1.8%	7.1%
	PMAD	73.9%	8.7%	7.5%	1.1%	1.8%	6.9%
	TOD100%	75.2%	7.8%	7.2%	1.1%	1.8%	6.9%
	Central	74.6%	8.1%	7.3%	1.1%	1.8%	7.2%

* Modal share reported without taking into account population weight. ** Bimodal: Car & PT; Car & bicycle; PT & bicycle.

**Table A2:** Modal share for each 2031 scenario, according to mode choice analyses and to our simpler method (weighted trips) *.

		Car	Public Transit	Walk	Bicycle	Bimodal **	Other ***
Mode choice analyses	BAU	62.5%	17.4%	12.5%	1.3%	1.6%	4.7%
	PMAD	66.8%	14.2%	10.6%	1.3%	1.6%	5.4%
	TOD100%	67.1%	14.1%	10.8%	1.2%	1.6%	5.2%
	Central	67.2%	14.0%	10.6%	1.2%	1.6%	5.4%
Simpler method based on 2008 mode shares	BAU	66.3%	14.5%	11.0%	1.3%	1.8%	5.1%
	PMAD	64.6%	15.5%	11.9%	1.5%	1.7%	4.9%
	TOD100%	63.4%	16.5%	12.4%	1.5%	1.7%	4.5%
	Central	59.9%	18.9%	14.0%	1.7%	1.5%	4.0%

* Weighted trips, represent the entire 2031 Greater Montreal Area population. ** Bimodal: Car & PT; Car & bicycle; PT & bicycle. *** Other: School bus or undetermined.
